# Workflow to Investigate Subtle Differences in Wine Volatile Metabolome Induced by Different Root Systems and Irrigation Regimes

**DOI:** 10.3390/molecules26196010

**Published:** 2021-10-03

**Authors:** Mani Awale, Connie Liu, Misha T. Kwasniewski

**Affiliations:** 1 Division of Plant Sciences, University of Missouri-Columbia, 135 Eckles Hall, Columbia, MO 65211, USA; maybd@mail.missouri.edu; 2 Department of Food Sciences, The Pennsylvania State University, 326 Rodney A. Erickson Food Science Building, University Park, PA 16802, USA; 3 Food Science Department, University of Missouri-Columbia, 135 Eckles Hall, Columbia, MO 65211, USA; liugw@missouri.edu

**Keywords:** rootstocks, untargeted metabolomics, features, grafted, multivariate analysis, aroma compounds

## Abstract

To allow for a broad survey of subtle metabolic shifts in wine caused by rootstock and irrigation, an integrated metabolomics-based workflow followed by quantitation was developed. This workflow was particularly useful when applied to a poorly studied red grape variety cv. Chambourcin. Allowing volatile metabolites that otherwise may have been missed with a targeted analysis to be included, this approach allowed deeper modeling of treatment differences which then could be used to identify important compounds. Wines produced on a per vine basis, over two years, were analyzed using SPME-GC-MS/MS. From the 382 and 221 features that differed significantly among rootstocks in 2017 and 2018, respectively, we tentatively identified 94 compounds by library search and retention index, with 22 confirmed and quantified using authentic standards. Own-rooted Chambourcin differed from other root systems for multiple volatile compounds with fewer differences among grafted vines. For example, the average concentration of β-Damascenone present in own-rooted vines (9.49 µg/L) was significantly lower in other rootstocks (8.59 µg/L), whereas mean Linalool was significantly higher in 1103P rootstock compared to own-rooted. β-Damascenone was higher in regulated deficit irrigation (RDI) than other treatments. The approach outlined not only was shown to be useful for scientific investigation, but also in creating a protocol for analysis that would ensure differences of interest to the industry are not missed.

## 1. Introduction

**Figure 1:**
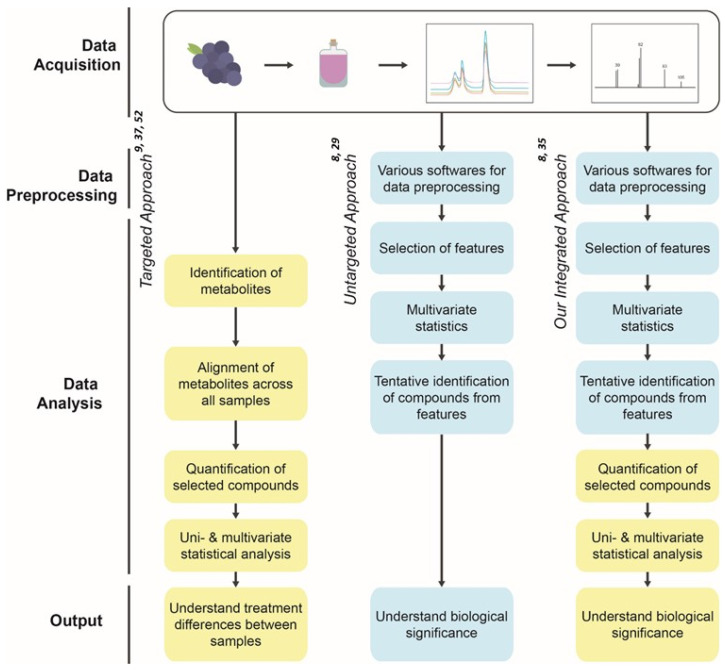
Comparative GC-MS based metabolomic approaches showing steps of conventional targeted approach, untargeted metabolomic approach and our integrated metabolomic approach that uses parts of both targeted and untargeted approaches.

Volatile composition plays a critical role in grape and wine quality and can capture information encompassing a year (or more) of vine growth in relation to its environment. Growing conditions (soil conditions, climate, temperature), vineyard management practices (irrigation, pruning, sun exposure) and vine genotypes, including both the scion and rootstock used, can all cause a cascade of metabolic shifts, some of which with direct impact on volatile compounds and others impacting the non-volatile metabolome which contributes to aroma precursors [1,2,3]. The process of wine-making and fermentation further impact metabolic processes, enhancing volatile metabolic differences in ways that may be complex and not intuitive [2]. To ensure all analytes of potential interest are included when studying such a complex system, using a metabolomics-based approach can have clear advantages by first starting with a bias towards inclusivity of more analytes rather than limiting characterization to just a few [4,5]. However, when relating metabolic shifts to bioactivity parameters (e.g., aroma perception), it is not enough to demonstrate a difference—it is also necessary to characterize the number of compounds present and the difference observed [6,7]. Additionally, in some metabolomic investigations, focus is first placed on those compounds that show the largest concentration difference as this indicates metabolic importance in aroma chemistry; this may miss critical changes in quality (Figure 1) [8,9]. This is why often, in flavor work, a quantitative, targeted approach is taken to ensure perception threshold and other elements where actual concentration can be used to interpret the data.

About 80% of all *Vitis vinifera* grapevines planted are grafted to rootstocks to protect against phylloxera [10,11], tolerance to biotic stresses such as nematodes [12] as well as tolerance to abiotic stress such as drought [13,14] and salinity. Grafting to rootstocks has been shown to impact the concentration of volatile compounds including esters, 2,3-butanediol [15], norisoprenoids and higher alcohols when compared to wines produced from own-rooted vines as well as across different rootstocks [16,17]. Water availability also can impact fruit and wine composition [18,19,20,21]. Moderate water stress mediated through deficit irrigation leads to reduced water uptake and reduced shoot growth and yield, leading to smaller berries with a higher concentration of aroma compounds in grapes and wine volatiles [17,19,22] and thus correlating with positive wine sensory attributes (more fruity and less vegetal) [23]. Rootstocks can alter plants’ ability to tolerate water stress, and the interaction between different irrigation and rootstocks may also impact the volatile profile of grapes and wines as water stress has been known to impact the wine volatiles [19,20,24,25].

Traditionally, the studies of the impact of rootstocks, irrigation and other viticultural management on the complex wine aroma and flavor were focused on a few important compounds including norisoprenoids and esters [17,20,22,25]. Recently, non-targeted metabolomics has been used in grape and wine studies to understand grapevine berry development, fungal pathogen in juice, comparison between wild and *vinifera* volatiles, wine classification, wine volatiles, non-volatiles, and unspecified compounds that are present in the wine as possible [4,5,26,27,28,29,30]. This approach was also used to study rootstock modulation of grape and wine aroma in Shiraz grapes, leading to the tentative identification of 152 impacted compounds [16]. This approach has particular value in uncharacterized and understudied cultivars as it can characterize complex general differences between populations and enable the identification of unknown compounds, or those not normally deemed as impertinent in wine aroma. Further, the volatile metabolome has value beyond just aroma compounds but also in understanding overall treatment effects.

Grapes with some non-*vinifera* parentage are known to have unique aroma characteristics. One such example is *V. labrusca* grapes, and hybrids of this species have methyl anthranilate and *o*-aminoacetophenone compounds, impacting odorants for these varieties that cause the characteristic foxy taste of these grapes which were not found to be present in *V. vinifera* grapes [31]. However, in many cases these clear impact odorants are not present, with it being hypothesized that what constitutes hybrid grape character is the sum of its complex aroma chemistry [32]. Given the great genetic diversity and unique odorants that can be found in North American species, it is clear that a targeted approach based on *V. vinifera* is likely to miss important compounds in hybrid grapes. On the other hand, an untargeted approach alone aiming at identifying the tentative compounds will not be sufficient as it lacks the ability to relate compounds to bioactivity and often overstates the influence of compounds at high concentrations.

Targeted analysis of specific metabolites has been traditionally used to understand wine aroma and to decouple the effect of different factors on fruit and wine quality. This approach involves identifying a selection of compounds from the chromatogram and quantifying those compounds in order to perform comparative analysis of the metabolic phenotypes using multivariate statistics (Figure 1). The advantage of this approach is that by designing analysis around those compounds to be identified and quantified, you can assure the overall method measures compounds in the ranges that have biological significance. The complexity of plant samples, including wine, presents multiple challenges such as coelution of metabolites and high variability in metabolic quantity within samples which results in reliable quantification due to method optimization for compounds of interest. However, this is done for only a limited number of metabolites [8]. Despite these limitations, it could be argued that this practice may be suitable in *V. vinifera* cultivars where important odorants have generally been identified. However, in understudied beverages, such as wines made from interspecific *Vitis* hybrid cultivars, a more all-encompassing approach such as untargeted metabolomics is essential to avoid missing important but not yet identified volatiles that could be critical to a wine’s overall character. This is all the more the case when considering that compounds are not perceived in isolation but rather components of the volatile metabolome may interact to impact perception. For example, recent work by Poivet et al. [33] demonstrates the interactions between odor active compounds, which would make measurements of a handful of compounds insufficient in characterizing aroma differences. The volatile metabolome is also not just of interest due to odor activity, but also demonstrates the impacts of all the factors outlined above, with grapes and other fruits acting as a “black box” whose information is released following fermentation.

An untargeted or metabolomics-based approach is comprehensive and unbiased. In this approach, spectral information from chromatograms is automatically transformed into coordinates based on mass, retention time, and associated signal intensity, which are then aligned across all samples to detect features or putative compounds without prior identification of the compounds. Various univariate and multivariate statistics are used to identify the metabolic features (*m/z* at the definite retention time) (Figure 1) [29,34,35]. However, this method also suffers from the limitations that it aims at identification of as many compounds as possible but often obtains relative differences without the quantitative data needed to relate to bioactivity or flavor. Similarly, unknown compounds makes the biological interpretation of the data even more challenging [8]. Both these approaches have benefits, however, due to complexity of plant systems; both of these approaches can also fall short especially in systems where subtle differences in trace compounds are important. This is often the case when investigating flavor as small differences in compounds below ng/mL level may have dramatic differences in quality or plant metabolic regulation, where organisms work to remain healthy and productive through pathways promoting equilibrium rather than dramatic shifts in chemistry [8].

To overcome the limitations of both the targeted and untargeted method, we developed an integrated metabolomics workflow that encompasses the benefits of both the approaches. Our workflow aims at untargeted data processing of the spectral data followed by various univariate and multivariate analyses to unbiasedly identify the compounds that are truly important in the experimental design. Our workflow then goes forward to integrate the components of the targeted approach where the significant compounds are confirmed with the authentic standards and then quantified so that the data is meaningful and interpretative in the real field situations (Figure 1).

## 2. Results and Discussion

An integrated metabolomics workflow was successfully used to find subtle differences in volatiles within rootstocks and irrigation treatments that would otherwise have been missed using traditional approaches. Targeted analysis of volatiles has been the standard for decades as it allows predictable quantification of selected compounds of interest, which in turn can be related to odor thresholds and can theoretically explain treatment impacts on aroma (Figure 1) [20,22,36,37,38,39,40]. This, however, assumes that all compounds of importance have been identified, as well as that odor thresholds are accurate (and immobile) [8]. Moreover, the complexity of plant samples poses problems such as compound coelution despite effective prior chromatographic separation that complicates their discrimination. While these assumptions may be accurate in well-studied plants and foods such as *V. vinifera*-based wines, poorly studied food such as wines made from interspecific hybrids benefit from a more holistic approach to data collection to avoid missing key analytes [29,41]. As such, it is appropriate to adopt inclusive initial analysis to avoid missing differences induced by the treatments, before deciding what compounds are of interest (Figure 1). The untargeted approach avoids the time-consuming need for the prior assignment of chemical information to the molecular structure for hundreds of datasets, which makes it faster and more unbiased [8]. This approach has proved invaluable in situations where stark differences are expected, such as comparing mutant and wild-type populations or infected and healthy individuals [5]. However, this type of analysis has not been widely used to characterize subtle differences that are inherent in a biological system, with researchers focusing on analytes with a 2-fold or higher change to ensure metabolic influence [41,42]. Usually, the variations in the metabolic phenotype in field conditions due to natural variability are not very contrasting in comparison to lab or greenhouse studies where all factors are controlled except the factor in study. Such phenotypes show subtle differences that need to be analyzed so that they are not overlooked. When dealing with a genetically identical grapevine scion (cv. Chambourcin in this case) grafted to genetically different root systems with three different irrigations, a hybrid integrated metabolomics approach that encompasses both the benefits of targeted and untargeted data analysis was used to allow inclusive characterization of even subtle differences on wine volatiles (Figure 1). This allowed for the comprehensive characterization of wine volatiles, prior to the laborious step of chemical identification, with quantitation and identification using authentic standards for only those compounds that are relevant to explaining treatment variation.

### 2.1. Untargeted Metabolomics Results

Data processing transforms the raw chromatographic data files into a format that is useable for further analysis which includes measurement of *m/z*, retention time of the ion, and ion intensity from each raw data file, which and are mostly done by softwares. Data processing includes removing noise, feature detection, alignment, retention time correction, and normalization. Using *XCMS online*, we identified a total of 682 and 877 metabolite features in 2017 and 2018, respectively (Figure 1), which is within the expected range of the number of features to be found in similar metabolomics studies [5,43]. While in many cases one compound can result in several features due to multiple ions making up its mass spectra, this still demonstrates a great increase in potential analytes over similar targeted studies of wine volatiles, with minimal front-end effort [44]. This step can be accomplished using other platforms such as PARADISe, MZmine 2, and OpenChrom; however, after extensive testing, we opted for *XCMS online* due to the wide array of chromatogram normalization and feature detection options, which facilitated including features at low concentrations for further statistical analysis.

**Figure 2:**
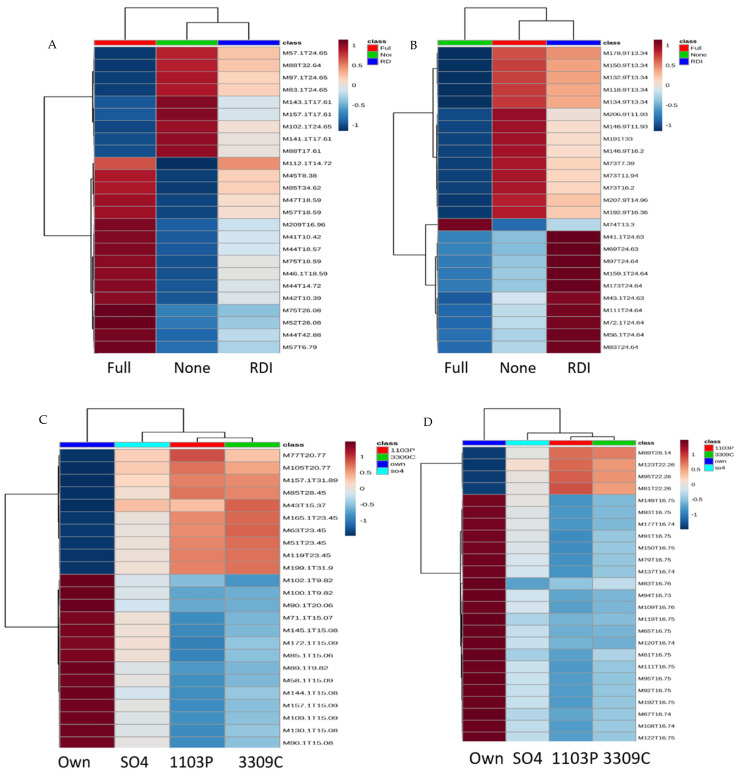
Heatmaps of the top 25 most influential features for differentiating wine volatiles by (**A**) irrigation regime in 2017, (**B**) irrigation regime in 2018, (**C**) root system in 2017, and (**D**) root system in 2018. While only the top contributors are shown, the heatmaps were generated using all features. The rows in the heatmap represent features (M(*m/z*)). T (time in minutes) and the columns indicate sample categories. The colors of the heatmap cells indicate the abundance of compounds across different samples. The color gradient, ranging from dark blue through white to dark red, represents low, middle, and high abundance of a compound.

Following feature identification, the next step in our integrated metabolomics workflow is statistical analysis of the detected features to identify features of interest, specifically finding those features impacted by the treatment. Data analysis for each year was performed separately as the analyses were conducted in different years, adding an instrumental effect to the raw data, and because vintage difference has been widely reported in previous studies as the largest influence in aroma variation [45,46,47]. Using ANOVA, 221 and 328 features were found to be significantly impacted by the root system (*p*_value_ < 0.05) (Figure 1, Supplemental Tables S1 and S3), and 380 and 85 features were found to be significantly impacted by various irrigation regimes in 2017 and 2018, respectively (*p*_value_ < 0.05) (Figure 1, Supplemental Tables S2 and S4). The root system and irrigation influence on wine features in both years is visualized using heatmaps, where the top 25 most significant features are shown as mean values within treatments (Figure 2). Both root system and irrigation had yearly differences in metabolites, feature grouping between treatments. In 2017, the top 25 wine features were similarly expressed in ‘RDI’ and ‘None’, whereas in 2018, wine metabolite features were similar between ‘Full’ and ‘RDI’ (Figure 2A). Between root systems, own-rooted vines had the strongest differences of the root systems for the top 25 metabolic features, contrasting them from all grafted vines (Figure 2C,D).

**Figure 3:**
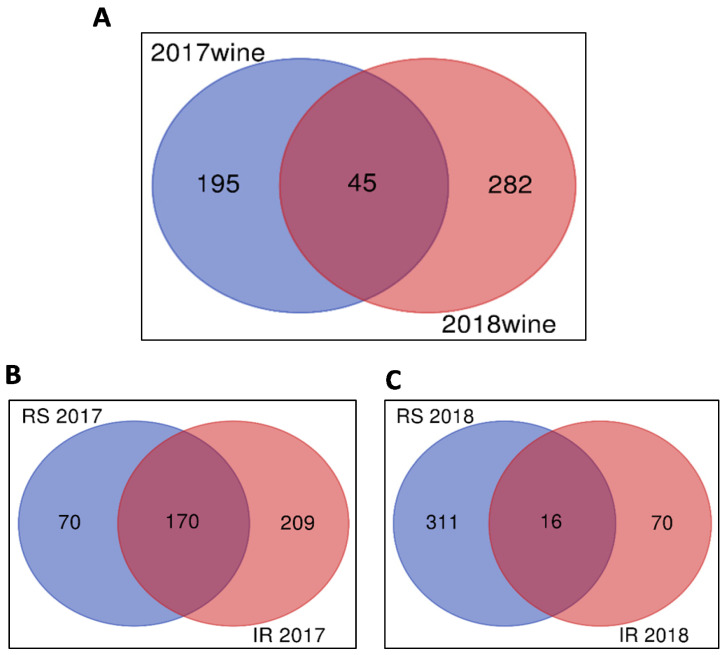
(**A**) Venn diagram showing common and unique features between wines in 2017 and 2018. Only 45 features were common in both years showing yearly differences in metabolites. (**B**) Venn diagram showing common and unique features between root systems and irrigation treatments in wines in 2017. 170 features were similar between root system and irrigation treatments. (**C**) Venn diagram showing common and unique features between root system and irrigation treatments in wines in 2018. Few features were similar between root system and irrigation treatments.

As the initial analysis was conducted separately for individual years to avoid instrumental impacts and to focus on treatment variation, the overlap between features was compared. We found 45 significant features that were common in 2017 and 2018 between root systems, as demonstrated by the Venn diagram (Figure 3A). This overlap was important to note as one of the limitations of untargeted analysis is the value in running all samples to be compared in one continuous set to avoid changes with the instrumentation driving the difference [29]. Despite the instrument being used at different times, a subset of features significantly explaining treatment effect were shared between years when the initial analysis was first grouped by analysis period (year) and then untargeted results compared across years. In an initial analysis of treatment effects, it was observed that a total of 170 and 16 features were common in 2017 and 2018, respectively, between root system and irrigation treatments (Figure 3B,C). This demonstrates that some shared metabolites were being significantly impacted both by irrigation and root system, whereas others were impacted solely by one or the other.

### 2.2. Identification of Significant Features

**Figure 4:**
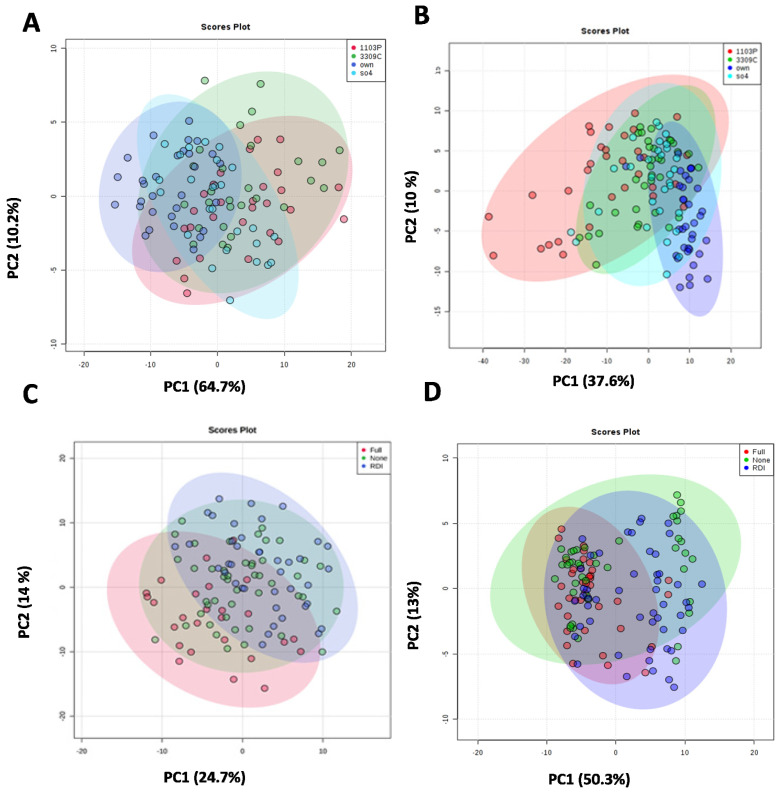
PCA scores plot of wine metabolite features separated by root system in (**A**) 2017 and (**B**) in 2018 regardless of the irrigation treatment. PCA scores plot of wine metabolite features separated by irrigation treatments in (**C**) 2017 and (**D**) 2018 regardless of the rootstock treatment. PCA was performed using log-transformed and autoscaled significant features.

Only those features found to be significantly influenced by treatment were included moving forward, as the ultimate goal was to identify those compounds impacted by treatments. Unsupervised principal components analysis (PCA) was performed to model significant features most important to treatment differences to determine patterns between multivariate samples. The impact of root systems on wine features can be observed in the PCA scores plot in which 38.4% and 10.6% of the total variance was explained by PC1 and PC2, respectively, in 2017 (Figure 4A). In 2018, PC1 explained 37.6% of the variance, and PC2 captured about 10% of the variance (Figure 4B). The PCs were also found to be significantly different from each other based on the ANOVA followed by Tukey’s honest significant difference (HSD) (*p*_value_ < 0.05). While there is not complete separation between root systems or irrigation treatments in both years, this partial separation suggests distinct patterns in at least some metabolite concentrations that may differentiate between the groups. However, further analysis was conducted to investigate this observation below. Such patterns may be weak due to the confounding effect of metabolites with strong variations due to other factors. This is often the case when modeling wine chemical differences; where even when including measurement shown to have some treatment influence, similarities across treatments will also be apparent in all but the most extreme cases [9,23,48,49]. A subtle but significant impact was also observed between rootstocks grafted to the same scion for many fruit composition and quality traits by [44], which reiterates the need for a careful holistic investigation when exploring subtle differences. Unlike in certain untargeted research, minute differences in chemistry can have large sensory and economic impacts despite being made from the same grape variety [50].

For a better understanding of the metabolic characteristics and interpretation of the results obtained by the unsupervised analysis model and to highlight the similarities and differences between treatments, the partial least squares-discriminant analysis (PLS-DA) method was applied. The PLS-DA analysis revealed the subtle separation between treatments. The PLS-DA models obtained were evaluated using leave one out cross-validation (LOOCV) where the quality of the fit was evaluated with *R*^2^ and the predictive capacity with *Q*^2^. In 2017, the PLS-DA model for the separation of root systems was *R*^2^ 0.37 and *Q*^2^ was 0.24 with two components. Similarly, for irrigation, *R*^2^ was 0.39 and *Q*^2^ was 0.14. In 2018, the *R*^2^ for PLS-DA model was 0.42 and *Q*^2^ was 0.11 for the classification of root systems, and *R*^2^ was 0.36 and *Q*^2^—0.18 for the PLS-DA model for classification between irrigation treatments (Supplemental Table S5). We obtained 153 and 125 features that have VIP scores >1 that cause separation between roots systems and irrigation, respectively, in 2017. In 2018, 150 and 146 features had VIP score >1 which showed that these features contributed to separation between treatments due to root systems and irrigations, respectively (Supplemental Table S6). The validation of the models was also performed through 1000 permutation tests, where the probability that the model was created by chance was less than 0.0001%, showing a level of confidence that the subtle separations are caused by the differences in the treatments. PLS-DA has been very commonly used in metabolomic studies to find the significant features that have treatment differences [8,42,51].

**Figure 5:**
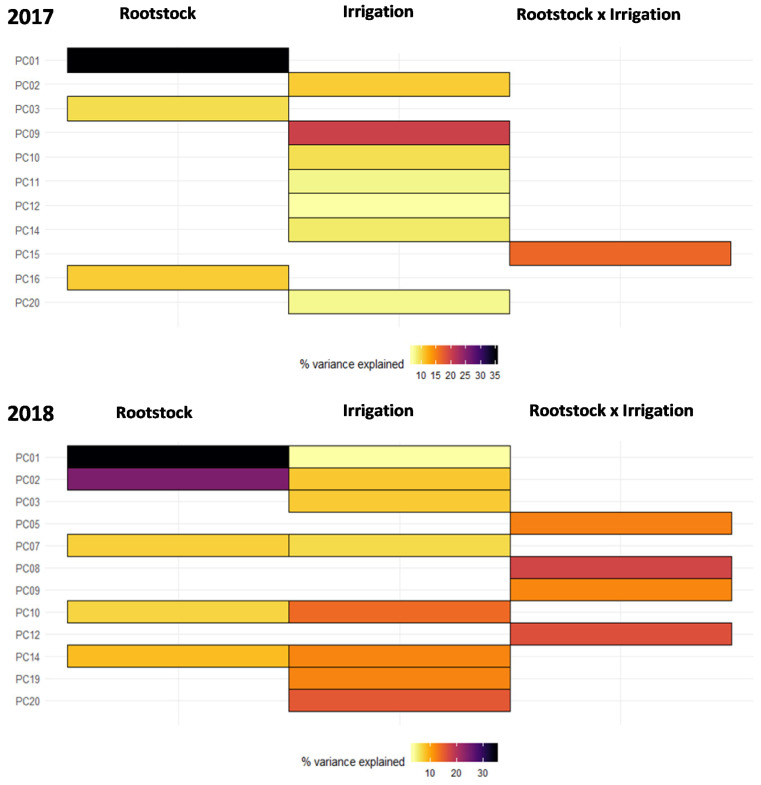
A linear model was estimated for wine 2017 and 2018 PCs 1 to 20. The model included rootstock (own-rooted, ‘1103P’, ‘3309C’, ‘SO4′), irrigation (Full, RDI, None), and rootstock by irrigation interaction. The 20 PCs capture 85.79% and 78.34% of the variance in 2017 and 2018. Only the factors which explained a significant portion of the variance (*p* < 0.05) are plotted. The percent variation explained by each factor is indicated using color.

As PC1 and PC2 could not explain the complete separation between treatments, a linear regression model was created that included root system (own-rooted, SO4, 1103P, and 3309C), irrigation (none, RDI, Full), and root system by irrigation interaction effect using the first 20 PCs. We observed that up to 35% of the variation in the root system was explained by a single PC (PC1) in 2017 (Figure 5A). Irrigation had a significant effect on PC2, PC9, PC10, PC11, PC12, PC13, PC14, and PC20, contributing up to 20% of the variation in wine volatiles (Figure 5A). Similarly, in 2018, root system contributed significantly to 5 PCs, explaining up to 35% of the variation, and irrigation contributing to 8 PCs explained significant variation up to 15% of variation by irrigation in 2018 (Figure 5B). We also observed some significant root system by irrigation interaction impact in both years as explained by PC15 (contributed 15% of variation) in 2017 (Figure 5A) and PC5, PC8, PC9, and PC12 (contributed up to 16% of variation) in 2018 (Figure 5B).

### 2.3. Compound Identification and Confirmation

Untargeted metabolomics data is most useful when the analytical signals (features) are used to identify metabolites or compounds and relate their intensities or concentrations to knowledge about the biological system. Using significant features from ANOVA and PCA loadings, identification of the compounds derived from the non-targeted analysis was conducted by comparing obtained mass spectra, at a definite time, with the NIST library, as well as by comparing the calculated retention index of tentatively identified compounds with that published by others. This two-way confirmation was crucial for the correct assignment of annotation of the compounds [51]. For example, in 2018, we identified *m/z* 69, 105, 121, 190 at retention time 21.73 to 21.82 min which was identified by the NIST library as β-Damascenone with 48% match probability. The calculated RI was 1820, which is close to that of literature (1832) [52]. A subset was further confirmed by comparison to authentic standards. Alternatively, compound identification can be confirmed using a second column type; however, this then necessitates duplicating all efforts to this point, including doubling the number of GC-MS runs [51]. We opted for confirmation via authentic standards as this would be needed for calibration curve generation. In this way, we first tentatively identified a total of 94 unique compounds in wines from 2017 and 2018 from the features that were significantly different due to root system and irrigation using their spectra, retention index and match score in the NIST library (Supplemental Table S7). There were many orphaned spectra that showed significance, which could not be identified with the NIST library. For example, in 2017, 11 spectra could not be definitively related to any compound. While further efforts could have been taken, we opted to prioritize those compounds with tentative identification given the large number still remaining.

### 2.4. Quantitation of the Compounds

To confirm tentative identification and quantify the compounds identified from the significant features (non-targeted analysis), we used authentic standards and an internal standard to generate calibration curves. For all compounds included in quantitative data linearity was calculated using a 1/x weighted regression, and all values were within the linear range (R2 0.99 or higher). The spectra and retention time of the tentative compound were matched with that of the authentic standard that was run using Agilent MassHunter Qualitative Analysis (Agilent Technologies, Palo Alto, CA, USA). In this way, we were able to confirm and quantify a subset of 21 and 22 compounds for 2017 and 2018, respectively, among the 94 compounds identified using features. Many compounds could not be confirmed due to lack of authentic standards, or several of the top matches in the NIST database having an erroneous match when compared with an authentic standard (a critical error that may not have been avoided with robust confirmation). This step was similar to traditional targeted analysis where the compounds are quantified or semi-quantified using a calibration curve of an authentic standard [51]. The benefit of taking the analysis through to this step was to obtain concentration information, which is critical in understanding what impact a compound has on the final wine aroma by calculating their odor threshold values. The approach of quantification after untargeted compound identification was also successfully adopted by Weingart et al. in 2011, where they were able to identify 63 metabolites and quantify 47 compounds [51].

**Figure 6:**
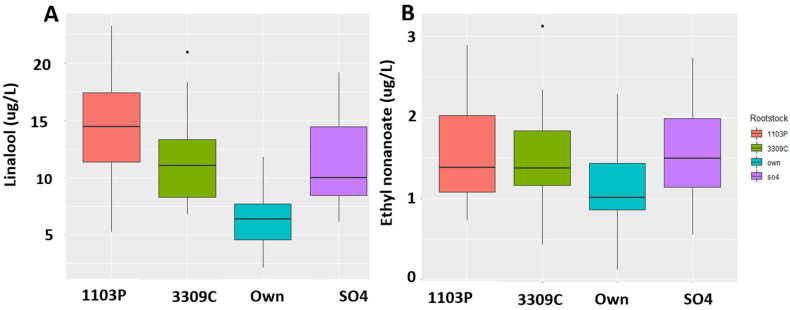
Boxplots of (**A**) β-Linalool (Linalool) and (**B**) Ethyl hexanoate concentrations differences in rootstocks and own-rooted Chambourcin. Rootstocks increased concentrations of Linalool and Ethyl nonanoate in wines 2018. Concentrations were measured in ppb (ug/L).

**Figure 7:**
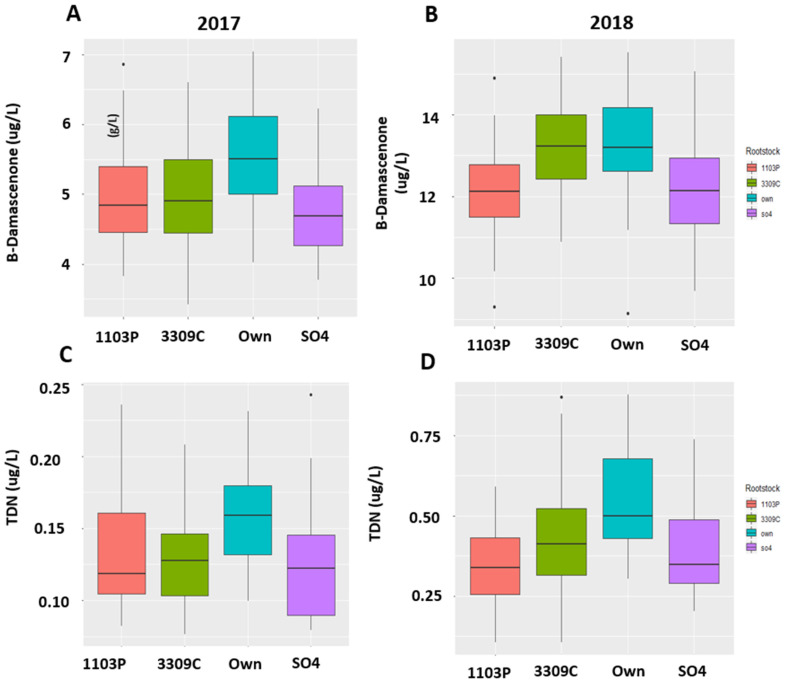
(**A**) Boxplots of β-Damascenone concentration differences in rootstocks and own-rooted Chambourcin in 2017 and (**B**) in 2018. (**C**) Boxplots of 1,1,6-trimethyl-1,2-dihydronapthalene (TDN) concentration differences in rootstocks and own-rooted Chambourcin in 2017 (**D**) in 2018. Both β-Damascenone TDN concentrations were found in higher in own-rooted Chambourcin in wines in both 2017 and 2018. Concentrations were measured in ppb (µg/L).

Using ANOVA (*p*_value_ < 0.05) for each compound followed by Tukey HSD, each compound was analyzed for significance based on both treatments and interaction as factors to make sure if the identified compounds were causing differences due to rootstocks as well as irrigation treatments. We found that the rootstock and irrigation interaction effect was significant for both 2017 and 2018 for several compounds (Table 1). We found 11 volatiles with significant rootstock and irrigation interaction effect in 2017, while Linalool was the only compound that showed significant rootstock and irrigation interaction effect in 2018 (Table 1). The compounds which were also significant in rootstock and the irrigation main effect are shown in Supplemental Table S7. Important wine volatiles such as β-Damascenone, Methyl Octanoate, Ethyl Nonanoate, and Linalool were significantly different between root systems in 2017. The wine volatiles that showed a significant difference between root systems at a 5% level of significance in 2018 are shown in Table S6. Generally, grafting Chambourcin vine to different rootstocks caused either an increase or decrease in volatile compounds in wines in both years. Linalool and Ethyl nonanoate were found to be higher in concentration in wines in rootstocks than own-rooted Chambourcin wines in 2018 (Figure 6), while wines from own-rooted vines showed higher concentration for β-Damascenone and TDN in both 2017 and 2018 (Figure 7).

A full list of compounds that are also found to be significantly different from irrigation treatments in 2017 in 2018 are presented in Supplemental Table S8. RDI resulted in a higher concentration of compounds in 2017 but not in 2018 (Supplemental Table S8). In 2018, not applying any irrigation led to an increase in the concentration of compounds (Supplemental Table S8). Deficit irrigation has been previously shown to increase volatile content in apples and grapes [53,54,55]. Water deficit activates the hydraulic and chemical signals (such as ABA) from the drying roots to the shoots that subsequently lead to reduced water use through decreased stomatal conductance through stomata closure. Deluc, Quilici, Decendit, Grimplet, Wheatley, Schlauch, Mérillon, Cushman, and Cramer [21], using metabolomics and transcriptomics, found that water deficit affected the ABA metabolic pathway in Cabernet Sauvignon and Chardonnay, with a high abundance of 9-cis-epoxycarotenoid dioxygenase (NCEDI) transcripts. The metabolic responses of grapes to water deficit varied with the cultivar, showing differences in ABA, isoprenoid, carotenoid, amino acid, and fatty acid metabolism.

We observed significant differences between own-rooted Chambourcin and Chambourcin grafted to rootstocks in many volatiles, which indicated that grafting is causing significant changes to the scion (Table 1). Such differences in volatile profiles of wines between own-rooted and rootstocks were also been observed in Shiraz [16] and Monastrell grapes [17]. Monastrell grapes grafted to different rootstocks and treated with deficit irrigation showed significant differences in aroma profile between rootstocks and irrigation. Being a vigorous rootstock, 1103P wines showed a higher concentration of aromatic compounds including alcohols, esters, and acetic acid than wines from other rootstocks. However, in that study, own-rooted Monastrell grapes were not included in the study. No significant interaction between rootstock and irrigation was observed. Wang, Chen, Gao, He, Yang, He, Duan, and Wang [38] found a higher concentration of total esters on own-rooted vines than on grafted vines in Cabernet Sauvignon.

The quantitative (and semi-quantitative) data from 2017 and 2018 differed from each other in concentrations (Supplemental Table S8). This is likely due to differences in growing conditions and environmental factors in both years that can significantly impact the aroma profile of grapes and wines. Analysis of the quantitative data of 2017 and 2018 showed a significant increase in concentrations of isoamyl acetate in wines regardless of treatments (Table 1). Fermentation results in many compounds in wine that are not seen in berries, created from berry precursors. Isoamyl acetate is an important ester derived during fermentation by *Saccharomyces cerevisiae* [56]. In 2017 Chambourcin wines, isoamyl acetate is present at a concentration of 495 µg/L, which is 16.5 times higher than the odor threshold, thereby showing the significance of this compound in the aroma of Chambourcin wines. This concentration was found to decrease in 2018 with an average concentration of 298.5 µg/L, which is about 10-fold higher than the odor threshold. Isoamyl acetate was found to be the compound responsible for the characteristic fermentation bouquet in Pinotage wine (average concentration 15.6 mg/L) and found to vary significantly from one vintage to another [56]. The synthesis of isoamyl acetate by *S. cerevisiae* during fermentation of wine had been found to be due to the activity of isoamyl alcohol acetyltransferases from the precursors present in the grapes, especially fatty acids [57].

By using the untargeted workflow, new compounds as well as compounds that were not known to have a significant impact due to treatments based on only targeted studies can be identified [51]. For example, in our study, isoamyl acetate was identified as one of the significant compounds contributing to Chambourcin aroma which was impacted by both irrigation and rootstocks. Generally, this compound was not identified and quantified as an important compound in irrigation and rootstock studies. Thus, our metabolomics-based approach was able to identify a compound that was known to be a fermentation compound but not usually studied as an important grape precursor-derived compound of significance in studies. Weingart, Kluger, Forneck, Krska, and Schuhmacher [51] also identified 19 metabolites that were not known in grape leaves using an approach that combined both untargeted and targeted approaches.

Some of the compounds that were known to be important to wine aroma and mostly included in targeted wine studies were also found using this metabolomics-based approach, for example, β-Damascenone. β-Damascenone is an important wine volatile, a C13-norisoprenoid formed from the degradation of carotenoids, and is known to increase with shading. This volatile was found higher in own-rooted vines (mean of 9.49 ug/L) than grafted vines (mean of 8.59 µg/L) in both years (Supplemental Table S8, Figure 7A,B). The decrease in vigor using low to medium vigor rootstocks (1103P, 3309C, and SO4) might have contributed to the lower concentration of this compound in vines grafted with rootstocks than high vigor own-rooted Chambourcin (Figure 7). Studies have also found an increase in β-Damascenone with the water stress which is consistent with the results of higher β-Damascenone concentration in wines from vines treated with RDI and None. The presence of this compound in all wines above the threshold (4–5 ug/L in wine matrix) demonstrated that this compound is a key aroma component in Chambourcin wines. Although this compound has honey, rose, and baked apple aroma, it has been known to impact the wine aroma by interacting with other aroma components. Thus, this metabolomics-based approach was useful to identify compounds that are significant for wine, including those that are known as well as unknown compounds.

Using a metabolomics-based approach followed by quantitation, we were able to identify subtle differences between rootstocks and irrigation in a hybrid cultivar Chambourcin grafted to three different rootstocks and own-rooted. This approach helped us to narrow down 800 features to 94 unique compounds using multivariate analysis. We confirmed and quantified 24 compounds in 2017 and 2018. In 2017, 12 compounds showed significant rootstock and irrigation interaction effect, whereas only one compound had a significant interaction effect between rootstock and irrigation in 2018. Rootstocks caused either increase or decrease of volatile compounds in wines in both years. In irrigation, RDI resulted in a higher concentration of compounds; however, yearly variation exists. Understanding the impact of rootstocks and irrigation on wine volatiles will prove useful in developing viticulture practices to manipulate grape aroma to produce wine with desired aroma quality. This metabolomics-based approach not only helps to identify and quantify compounds that are known to be important in wine but also those that are lesser-known but significant.

## 3. Materials and Methods

### 3.1. Study Design and Sampling

The samples were collected from an experimental vineyard at The University of Missouri Southwest Center, Mount Vernon, Missouri in 2017 and 2018. The experimental vineyard consisted of cv. Chambourcin (a red-skinned interspecific hybrid) scions grafted onto three different commercial rootstocks: 1103P, 3309C, S04 as well as non-grafted, own-rooted Chambourcin (‘3309C’- *V. riparia x V. rupestris;* ‘1103P’-*V. berlandieri x V. rupestris*; ‘SO4′- *V. berlandieri x V. riparia*). In addition to the four different root systems, three irrigation regimes were implemented in a full factorial design with irrigation treatments including full irrigation, regulated deficit irrigation (RDI), and no irrigation (full compensation of evapotranspiration losses (ET), 50% compensation of ET, and non-irrigated, respectively). Among the nine rows used in the study, each row had a different randomly assigned irrigation treatment and consisted of four 4-vine rootstock blocks randomly ordered with two guard vines at either end of a study row. For this study, only the middle two vines (vine 2 and 3) were sampled from each block. Irrigation treatments were initiated when water stress was observed, usually several weeks before veraison as the site has ample spring precipitation. The fruit was harvested in 2017 and 2018 from the 71 plants (9 rows*4 blocks*2 vines) individually into separate bins. Vine 2 in row 10 reverted to its SO4 rootstock, so this rootstock vine was not included in the analysis. More information about the vineyard design can be found at [58,59].

### 3.2. Winemaking

The wine was made on a per vine basis. Grapes were harvested in relatively similar time post veraison and with similar brix rate in both years (average of 22.79 and 20.6 in 2017 and 2018, respectively, with standard deviation of 0.92 and 0.63). Grapes from all vines were harvested on the same day, irrespective of the maturity stage of the vines. Fruit from each vine was separately harvested and transported to the winery at the University of Missouri. The grapes were stored in a cold room at 4 °C overnight and processed the day following harvest. The grapes were crushed and destemmed using Enoitalia destemmer-crusher (Cerreto Guidi, Italy). Sulfur dioxide (50 mg/L total) was added to each fermenter immediately after the crush, with inoculation occurring approximately 12 h later. The fermentations were carried out in 1-gallon fermentation vessels equipped with airlocks. Must was inoculated with GRE yeast (Lallemand, Petaluma, CA, USA) at the rate of 1 g/L on day 2 and rehydrated with Go-Ferm yeast nutrient (Lallemand) according to the manufacturer’s rehydration protocol. Go-Ferm was added to confirm healthy yeast hydration, and no other additions were made in the winemaking process. The ferments were punched down twice a day for 10 days, at which point they were pressed based on the clinitest. Malolactic fermentation was not initiated. The wines were racked approximately 21 days after pressing. To decrease the headspace, marbles were added to containers as needed as well as any headspace purged with nitrogen gas. The wines were filtered and bottled in 355 mL amber bottles. Following bottling, all wines were stored at 4 °C until analysis. Wines from 2017 were analyzed two years after bottling, whereas in 2018, wines were analyzed one year after bottling.

### 3.3. Reagents and Chemicals

All aroma standards other than 1,1,6-trimethyl-1,2-dihydronapthalene (TDN) were purchased from Sigma-Aldrich (St. Louis, MO, USA) at >98.8%. TDN was donated from Dr. Gavin Sack’s lab at Cornell University, which had been synthesized from α-ionone (Sigma-Aldrich, 99%) via ionene [60]. A C7-C30 hydrocarbon mixture, used for the determination of Kovat’s retention indices, was obtained from Sigma-Aldrich. Sodium chloride was purchased from Fisher Chemicals (Fair Lawn, NJ, USA). Ultrapure water (Type 1 water) was prepared using the ELGA Lab Water PURELAB Classic (High Wycombe, UK). L-Tartaric acid (99%) was obtained from Sigma-Aldrich.

### 3.4. Extraction of Wine Volatiles

In a 15 mL amber glass vial with screw cap, 5 mL wines were spiked with 50 µL of the internal standard solution to yield a final internal concentration of 0.05 mg/L (50 ppb) 2-Octanol, 0.1 mg/L (100 ppb) of 4-methyl-2-pentanol, and 0.05 mg/L (50 ppb) 3-Octanone. These compounds are not found naturally in wine and are chemically similar to compounds that are present in wine. While 2-Octanol was used to standardize response for initial metabolomic analysis, the other two internal standards were used for quality control. To the sample vials, 2 g of NaCl was added to inactivate the enzymes to improve headspace partitioning [37]. The glass vials were sealed and then loaded into the GC-MS/MS where the samples were processed for volatile aroma compounds using the HS-SPME-GC-MS/MS method outlined below. All 71 samples were run in duplicate in randomized order in two sequential orders, and internal standards were added for both metabolomic and quantitative analyses to ensure all conditions remained comparable throughout the experiments. Blanks were run after every 5–6 runs to prevent sample carryover.

### 3.5. HS-SPME-GC-MS/MS

In 2017 and 2018, semi-quantitative analyses were conducted using the triple quadrupole in a scan or using MRM mode for some compounds as needed. A 65 μm PDMS/DVB 1 cm SPME fiber, 23 gauge, coated with Polydimethylsiloxane-divinylbenzene-carboxen (PDMS/DVB 65 μm; Supelco) was used for sampling and extraction [61]. Fibers were conditioned before use according to the manufacturer’s recommendations. Wine samples in 15 mL sample vials were pre-incubated for 15 min at 45 °C. The fiber was exposed for 45 min at 45 °C in the headspace for volatile extraction. Samples were agitated in autosampler incubator at 500 rpm during extraction.

The HS-SPME GC-MS/MS system consisted of a MicroCal autosampler (MicroCal, LLC, Northampton, MA, USA) mounted on an Agilent 7890A gas chromatograph (Santa Clara, CA, USA) coupled with an Agilent 7000 Triple Quadrupole detector. The 65 μm PDMS/DVB SPME fiber was desorbed in the inlet at 250 °C for 2 min in splitless mode (inlet glass liner/SPME direct, 0.75, I.D., Supelco), after which the split flow was turned on (50 mL/min) for the remainder of the GC-MS run; the SPME fiber was conditioned in the inlet for 14.7 min before it was inserted into the next sample. No carry-over was observed between samples. Seventy-one wine samples, in duplicate, were analyzed in random order in two sequential orders with the features averaged. To prevent carry over of samples, a blank was run after every 4 or 5 samples. A DB-WAXetr column (30 m × 0.25 mm ID., 0.25 μm film thickness; Agilent Santa Clara, CA, USA) and helium carrier gas (flow rate: 1.2 mL/min) were used for all analyses. The GC oven temperature program was as follows: the initial temperature was 40 °C for 1.0 min then was increased to 200 °C at 5 °C/min followed by a second increase at 12 °C/min to the final temperature of 240 °C, which was held for 10 min [61]. For GC-MS/MS, the temperature of the transfer line was 240 °C, and nitrogen (1.5 mL/min) was used as the collision gas. The mass spectrometer was operated in electron ionization mode at 70 eV with multiple reaction monitoring (MRM) for quantification, with the monitored transitions (β-Damascenone 190–121 *m/z*; p-Cymene 134–119 *m/z*; Terpinolene 136–121 *m/z*; β-Linalool 136–93 *m/z*; TDN 157–142 *m/z*; Methyl Salicylate 152–120 *m/z*; α-Terpineol 136–121 *m/z*; Caryophyllene 201–189 *m/z*; β-Ionone 192–177 *m/z*; Ethyl dihydrocinnamate 178–104 *m/z*). Data acquisition and qualitative analyses were performed using the MassHunter Workstation software version B.07.00 (Agilent Technologies).

### 3.6. Data Processing Using Untargeted Metabolomics Analysis

Data collected by scan mode were processed using XCMS Online [35,62] The raw chromatographic data files (.D format) acquired by MassLynx from GC-MS were converted to .mzML data using the msconvert tool from ProteoWizard [63]. The data files (.mzML files) were uploaded to XCMS online, and each year’s data was processed as a single job for peak detection, retention time correction, chromatogram alignment, metabolite feature annotation, statistical analysis, and putative identification using the default parameters (feature detection: centWave method, min. and max. peak width = 5 and 20, S/N thresholds = 6, mzdiff = 0.01, integration method = 1, prefilter peaks = 3, prefilter intensity = 100, noise filter = 0, retention time correction: OBI-Warp method, profStep = 1; alignment: mzwid = 0.015, minfrac = 0.5, bw = 5, max = 100, minsamp = 1) [35,62]. Results were downloaded from the XCMS online on June 11–12, 2019. The extracted features (intensity of a given *m/z* at a certain time) were used for further analysis in defining treatment differences. The features that are significant for rootstock or irrigation using ANOVA (FDR adjusted *p*_value_ < 0.05) [64] were filtered and used for further analysis. Principal component analysis (PCA) applied after unit variance (UV) was evaluated for sample discrimination. Ellipses were used to represent 95% confidence interval. PLS_DA analysis was also performed using MetaboAnalyst 4.0 (Montreal, Canada) [65].

### 3.7. Identification and Confirmation of the Compounds

After identification of significant features using ANOVA and PCA loadings, the significant features were grouped based on their retention time. The compounds represented by the features were tentatively identified using the NIST MS Search v2.2, NIST 14 Mass Spectral Library database (Scientific Instrument Services, Ringoes, NJ, USA) by matching the mass spectral data with that of the compound. Only the compounds that had high match score (over 700) to the NIST database were considered. Additionally, linear retention indices (RI) were calculated using Kovats’ equation from a sequence of linear hydrocarbons from C7 to C30 to verify the NIST match with that of literature. Thus, two-step identification was made for the volatile compounds as possible matches were first identified by comparison of the mass spectral data within the NIST library and then verified as a valid prospect based on RI data.

The confirmation and quantitation of volatile compounds were achieved using calibration curves for each standard at five different concentration levels in cases where standards were available. For compounds whose standards were not available, semi-quantitative analysis was done by assuming a response factor equal to 1- to 2-Octanol IS equivalents. Agilent MassHunter Quantitative Analysis (for QQQ) B.07.01 (Agilent Technologies) was used. Each standard was prepared in model wine solution (8 g/L of tartaric acid, dissolved in 13% ethanol solution (*v*/*v*), at pH 3.2, adjusted with NaOH). Due to a large number of features and potential compounds (Supplemental Tables S1–S3), a further level of filtering was needed to keep authentic standard costs to a reasonable level. Odor activity value was chosen as a way to ensure priority was given to compounds likely to impact wine quality rather than just metabolic shifts of unknown importance. Odor thresholds of compounds found in the literature were used to calculate their odor activity values which show the relative contribution of each volatile compound to the final aroma of the wine.

## Figures and Tables

**Table 1:** Mean concentrations of wine volatiles in 2017 and 2018 between rootstocks and irrigation.

	2017												
Compounds	Full	Full	Full	Full	RDI	RDI	RDI	RDI	None	None	None	None	*p-Value*
	1103P	3309C	SO_4_	Own	1103P	3309C	SO_4_	Own	1103P	3309C	SO4	Own	
**Isoamyl acetate**	407.1 b ^1,2^	1080.8 a	391.2 b	381.8 b	559.5 b	553.2 b	431.5 b	244.9 b	449.2 b	573.5 b	483.4 b	386.0 b	*0.0203*
**Ethyl heptanoate**	397.7 c	295.1 cd	422.7 c	589.5 ab	203.1 d	443.0 bc	367.9 c	640.7 a	363.8 c	352.7 c	366.3 c	583.2 ab	*0.030*
**Ethyl octanoate**	38.3 c	26.7 cd	44.7 bc	67.1 a	15.6 d	44.3 bc	38.0 c	76.6 a	37.9 c	35.1 cd	37.3 c	61.1 ab	*0.040*
**1-Octanol**	15.8 cd	15.7 cd	14.5 cd	14.3 cd	19.4 a	15.9 cd	16.3 bcd	13.6 d	14.9 cd	16.7 bc	18.7 ab	14.0 cd	*0.000*
**1-Nonanol**	12.3 ab	13.2 ab	12.6 ab	12.3 ab	14.5 a	12.3 ab	14.7 a	11.0 b	12.7 ab	15.2 a	12.9 ab	12.5 ab	*0.100*
**Ethyl hydrocinnamate**	3.0 bc	2.5 bcde	1.9 cdef	1.6 def	4.7 a	2.6 bcde	2.7 bcd	1.4 f	2.7 bcd	2.2 bcdef	3.2 b	1.5 ef	*0.000*
**1-Dodecanol**	0.4 abcd	0.3 bcd	0.4 abc	0.5 ab	0.3 d	0.4 abcd	0.3 bcd	0.4 abc	0.5 a	0.3 d	0.4 abcd	0.5 ab	*0.030*
**Ethyl-tetradecenoate**	42.6 bc	46.6 bc	46.6 bc	52.2 bc	56.8 ab	69.3 a	48.3 bc	36.9 c	55.2 ab	44.7 bc	55.9 ab	40.8 bc	*0.000*
**Ethyl hexadecanoate**	192.4 bc	168.6 bc	186.5 bc	234.1 abc	249.0 ab	317.9 a	222.5 bc	153.1 bc	249.5 ab	145.2 c	238.8 abc	185.2 bc	*0.000*
**Isoamyl hexanoate**	1.6 bc	1.5 bc	1.4 cd	1.3 cd	2.3 a	1.8 b	1.6 bc	1.0 d	1.5 bc	1.9 b	1.8 b	1.24cd	*0.000*
**2-Phenylethyl acetate**	1.6 bc	35.2 bcd	28.6 cde	28.2 cde	60.4 a	41.8 bc	39.8 bcd	15.7 e	35.2 bcd	46.70 b	39.1 bcd	24.9 de	*0.000*
	**2018**												
**Linalool**	15.6 a	11.5 bcd	9.7 cd	8.4 de	16.1 a	10.6 bcd	11.4 bcd	5.6 e	11.5 bcd	12.1 bc	13.4 ab	6.1 e	*0.005*

^1^ Values represent μg/L concentration generated by comparing the peak area relative to the internal standard (2-Octanol) and comparing to calibration curve generated with an authentic standard. ^2^ Different letters within a row indicate significant differences for Duncan’s Multiple Range test at *p* < 0.05 among the rootstock irrigation interaction effect. Analysis of variance was used to compare data with Rootstocks, Irrigation and Rootstock × Irrigation as factors.

## Data Availability

The raw data supporting this article will be made available upon request from the corresponding author.

## Supplementary Materials

The following are available online, Table S1: List of the metabolic features (*M/z*, RI) identified from XCMS online that are significantly different between rootstocks in wines in 2017, Table S2: List of the metabolic features identified from XCMS online that are significantly different due to irrigation treatments in wines in 2017, Table S3: List of the metabolic features that are significantly different between rootstocks in 2018, Table S4: List of the metabolic features that are significantly different due to irrigation treatments in 2018, Table S5: Features with VIP scores >1 showing differences due to root systems in 2018, Table S6: Cross-validation details for the PLS-DA analysis for the root system differences in 2018, Table S7: Compounds tentatively identified by spectral comparison and retention index in wines 2017 and 2018 that had significant differences by area because of irrigation and rootstock treatments, Table S8: Concentrations of wine volatiles quantified (μg/L(ppb)) in 2017 and 2018 using GC-MS/MS; the *p*-values for significance for rootstocks and irrigations are also shown.

## Sample Availability

Samples of the grapes and wines are available from the authors.
